# Silencing repetitive DNA

**DOI:** 10.7554/eLife.29503

**Published:** 2017-08-01

**Authors:** Nahid Iglesias, Danesh Moazed

**Affiliations:** 1 Howard Hughes Medical Institute, Department of Cell Biology, Harvard Medical School, Boston, United States; 1 Howard Hughes Medical Institute, Department of Cell Biology, Harvard Medical School, Boston, United Statesdanesh@hms.harvard.edu

**Keywords:** chromatin, heterochromatin, non-coding RNA, histone methylation, Human, Mouse

## Abstract

Some RNAs in mammalian cells can help to silence the DNA they are transcribed from.

**Related research article** Johnson WL, Yewdell WT, Bell JC, McNulty SM, Duda Z, O'Neill RJ, Sullivan BA, Straight AF. 2017. RNA-dependent stabilization of SUV39H1 at constitutive heterochromatin. *eLife* **6**:e25299. doi: 10.7554/eLife.25299

**Related research article** Velazquez Camacho O, Galan C, Swist-Rosowska K, Ching R, Gamalinda M, Karabiber F, De La Rosa-Velazquez I, Engist B, Koschorz B, Shukeir N, Onishi-Seebacher M, van de Nobelen S, Jenuwein T. 2017. Major satellite repeat RNA stabilize heterochromatin retention of Suv39h enzymes by RNA-nucleosome association and RNA:DNA hybrid formation. *eLife* **6**:e25293. doi: 10.7554/eLife.25293

**Related research article** Shirai A, Kawaguchi T, Shimojo H, Muramatsu D, Ishida-Yonetani M, Nishimura Y, Kimura H, Nakayama J-I, Shinkai Y. 2017. Impact of nucleic acid and methylated H3K9 binding activities of Suv39h1 on its heterochromatin assembly. *eLife* **6**:e25317. doi: 10.7554/eLife.25317

Some of the DNA within our cells is packaged into a dense structure known as heterochromatin, which is often thought of as the dark matter of the genome because it effectively 'silences' regions of DNA that are potentially harmful to cells. In mammals, the bulk of heterochromatin forms at repeated DNA sequences called satellite repeats, which are found near a region of the chromosome known as the centromere (Figure 1A; Saksouk et al., 2015). However, it is also found at repeated DNA sequences near the ends of chromosomes and at mobile DNA elements known as transposons, which are interspersed throughout the genome.

**Figure 1.**
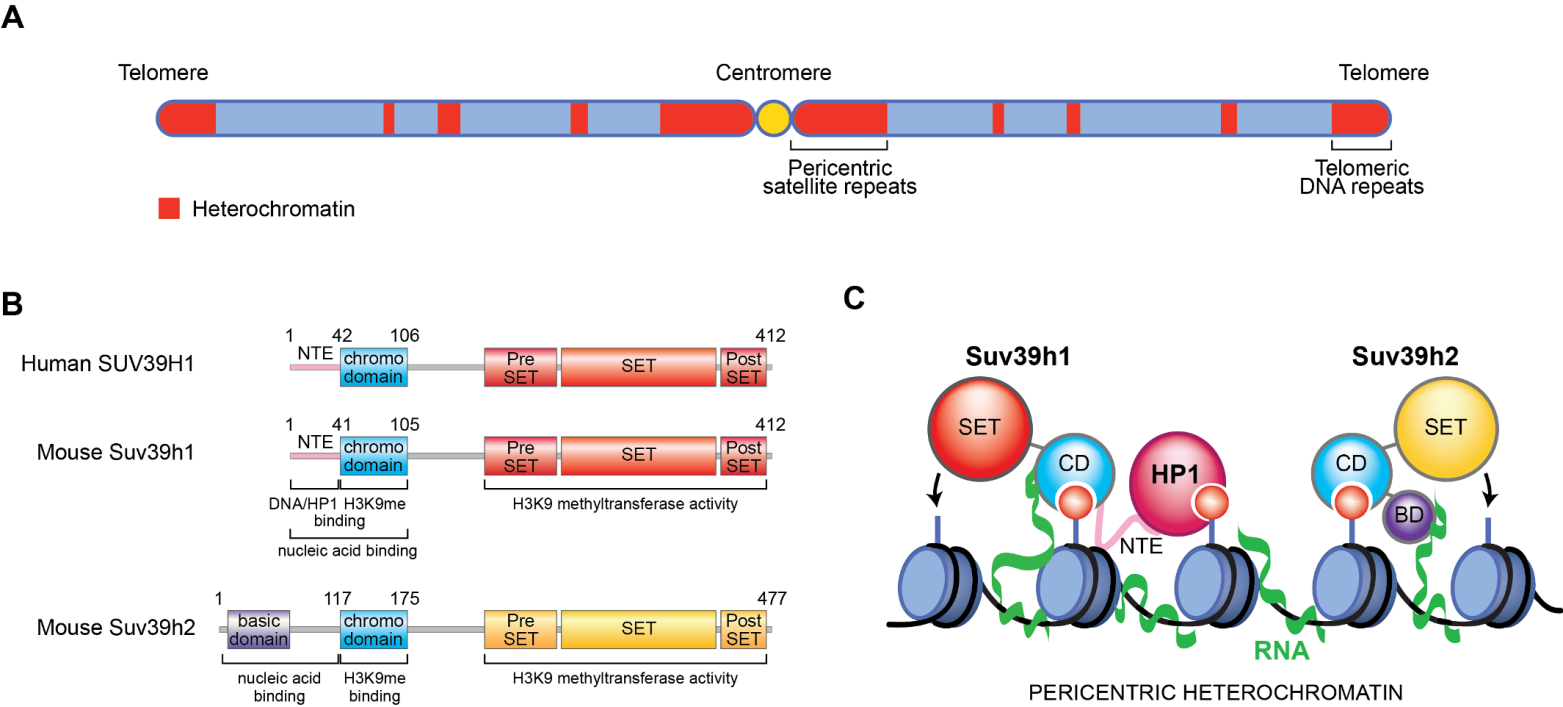
New role for RNA in retaining Suv39h enzymes on heterochromatin. (**A**) Mammalian chromosomes generally have several regions where DNA is tightly packed into a structure called heterochromatin (red). These include repeated DNA sequences near to centromeres (called pericentric satellite repeats) and other DNA repeats at the ends of chromosomes (called telomeric DNA repeats). (**B**) A human Suv39h enzyme called SUV39H1 and two mouse enzymes (Suv39h1 and Suv39h2) all contain a chromodomain (CD; turquoise) and a SET domain (shown in red and yellow), which can add methyl groups to a specific location on histone H3. Suv39h2 also has a basic domain (BD; purple) at the N-terminal end of the protein, while the other two enzymes have a region known as the N-terminal extension (NTE; pink). (**C**) Johnson et al., Shirai et al., and Velazquez Camacho et al. found that H3K9me3 modifications (small red circles) on histones (blue) and noncoding RNA (green) transcribed from pericentric satellite repeats work together to promote the association of mouse Suv39h1 (left), Suv39h2 (right) and human SUV39H1 (not shown) with heterochromatin. For Suv39h1, different surfaces on the chromodomain are involved in binding to H3K9me3 modifications and RNA, while the NTE interacts with DNA (black) and a downstream factor known as heterochromatin protein 1 (HP1), which is required to silence DNA. For Suv39h2, the basic domain and the chromodomain interact with RNA and H3K9me3, respectively.

The DNA in chromosomes is wrapped around proteins called histones. To make heterochromatin, enzymes of the Suv39h family modify the H3 histone by adding methyl groups to a particular location (to produce a modification known as H3K9me3). Proteins containing a region known as the chromodomain are able to bind to this H3K9me3 mark. This, in turn, leads to the recruitment of downstream factors that prevent the DNA being transcribed to make RNA molecules.

Over the past two decades, studies in fission yeast, plants and various animals have identified a role for RNA molecules that do not encode proteins and proteins that bind to RNA in the recruitment of Suv39h enzymes to heterochromatin (Holoch and Moazed, 2015). Many of these noncoding RNAs appear to be involved in a process known as RNA interference (RNAi), in which small RNA molecules reduce the activity of specific regions of DNA.

In flies and mammals, RNAi seems to be only required for silencing DNA repeats in germline cells (Aravin et al., 2007). Some studies have found that other noncoding RNA molecules acting independently of RNAi can also have silencing roles (Holoch and Moazed, 2015). However, it was not known whether noncoding RNAs transcribed from DNA repeats had a role in the formation of heterochromatin in non-germline cells in animals. Now, in eLife, three independent studies report that RNAs bound to DNA near centromeres allow mammalian Suv39h enzymes to stay attached to heterochromatin for longer periods of time (Johnson et al., 2017; Shirai et al., 2017; Velazquez Camacho et al., 2017).

Previous work has shown that the fission yeast homolog of the mammal Suv39h family can directly bind to RNA and DNA in cell-free systems through a region of the enzyme known as the chromodomain (Ishida et al., 2012). One member of the Suv39h family in humans, known as SUV39H1, is known to directly bind to RNAs associated with telomeres (regions at the very end of chromosomes) via its chromodomain (Porro et al., 2014). Furthermore, it has been reported that a noncoding RNA targets mouse Suv39h1 to a specific gene expressed in stem cells (Scarola et al., 2015).

Aaron Straight of Stanford University and co-workers at various universities in the US – including Whitney Johnson and William Yewdell as joint first authors – set out to determine whether RNA is associated with heterochromatin in human cells. They found that RNAs transcribed from DNA repeats called α-satellites near centromeres remain where they are made and co-localize with the H3K9me3 heterochromatin mark on chromosomes in cells that are preparing to divide (Johnson et al., 2017).

Subsequently, Johnson et al. observed that treating these cells with drugs that block transcription, or an enzyme that degrades single-stranded RNAs, results in fewer SUV39H1 enzymes being associated with the chromosomes. They also found that SUV39H1 could bind to both RNA and DNA (without sequence specificity) through its chromodomain and another region called the N-terminal extension (Figure 1B).

To explore the underlying mechanisms in more detail, Johnson et al. generated mutant versions of SUV39H1 that were unable to bind to DNA and RNA, but were able to interact with and modify the histone H3 protein. Adding further mutations that disrupt the ability of the chromodomain to recognize H3K9me3 modifications showed that SUV39H1 needs to bind to both H3K9me3 and RNA or DNA in order to form stable associations with heterochromatin and efficiently silence α-satellite repeats.

In the second study, Yoichi Shinkai at RIKEN, Jun-ichi Nakayama at the National Institute of Basic Biology and co-workers – including Atsuko Shirai and Takayuki Kawaguchi as joint first authors – at various institutes in Japan showed that the chromodomain of Suv39h1, the mouse homolog of human SUV39H1, directly binds to RNA (and also to DNA, but with a lower affinity) with minimal sequence specificity (Shirai et al., 2017; Figure 1B). Suv39h1 enzymes with mutations in the chromodomain that make them unable to bind to RNA or DNA, but do not affect other features of the enzymes, were less able to associate with heterochromatin near centromeres.

Shirai et al. also found that the mutant Suv39h1 enzymes were less likely to associate with RNAs produced by satellite DNA in mice. Decreasing the levels of major satellite RNAs in the cells appeared to reduce the ability of Suv39h1 to associate with heterochromatin near the centromeres. Thus, similar to what Johnson et al. say for the human version of the enzyme, Shirai et al. found that the chromodomain of murine Suv39h1 is able to bind to RNA and DNA, which cooperates with its H3K9me3 binding activity to allow it to stay attached to heterochromatin (Shirai et al., 2017).

In the third study, Thomas Jenuwein at the Max Planck Institute of Immunobiology and Epigenetics and co-workers – including Oscar Velazquez Camacho as first author – uncovered a basic domain at the N-terminal end of another mouse Suv39h enzyme known as Suv39h2 that is able to bind to RNA (Velazquez Camacho et al., 2017). This domain, which is absent from Suv39h1 (Figure 1B), binds specifically to long single-stranded RNA molecules, such as telomeric RNAs and the RNAs produced by satellite DNA near centromeres, but it does not bind to DNA. In mice, the N-terminal region helps Suv39h2 to associate with heterochromatin near the centromeres of cells that are preparing to divide.

Using a biochemical assay, Velazquez Camacho et al. – who are based at institutes in Germany, the Netherlands and Turkey – showed that most major satellite RNA is stably bound to chromatin and may form the 'scaffolds' that are required for Suv39h enzymes to remain associated with purified DNA wrapped around histones. However, expressing a mutant Suv39h2 enzyme that does not contain the N-terminal extension in cells that were missing the wildtype Suv39h1 and Suv39h2 enzymes restored the H3K9me3 modifications and the silencing of major satellites to nearly normal levels. This suggests that the possible biological role of Suv39h2 in RNA binding may be masked by redundant mechanisms.

Together, these studies suggest a new role for noncoding RNAs transcribed from DNA repeats, a role that involves helping Suv39h enzymes to associate with heterochromatin. The three teams all propose that the RNAs provide tethers that work together with histone H3K9me3 modifications to stabilize the binding of Suv39h enzymes to heterochromatin, leading to further H3K9me3 modifications and the silencing of DNA (Figure 1C).

Although the latest work supports a role for non-specific RNA binding in the tethering of Suv39h enzymes to heterochromatin, a role for DNA binding cannot be ruled out. In particular, a recent study suggests that the N-terminal extension of the human SUV39H1 enzyme promotes interactions with purified DNA wrapped around histones and stimulates H3K9me3 modification (Müller et al., 2016).

More generally, noncoding RNAs have also been implicated in the association of other histone-modifying enzymes with DNA in chromosomes. Like the members of the Suv39h family discussed here, these enzymes are able to bind to a variety of RNAs in cell-free systems. Therefore, the findings of these three studies may be applicable to other enzymes that control the way DNA is packaged in chromosomes.
